# Shared component modelling as an alternative to assess geographical variations in medical practice: gender inequalities in hospital admissions for chronic diseases

**DOI:** 10.1186/1471-2288-11-172

**Published:** 2011-12-21

**Authors:** Berta Ibáñez-Beroiz, Julián Librero-López, Salvador Peiró-Moreno, Enrique Bernal-Delgado

**Affiliations:** 1 Centro de Investigación Biomédica (CIB). Navarra, Spain; 2 Instituto de Investigación Sanitaria Aragón. Zaragoza. Spain; 3 Centro Superior de Investigación en Salud Pública (CSISP). Valencia. Spain

## Abstract

**Background:**

Small area analysis is the most prevalent methodological approach in the study of unwarranted and systematic variation in medical practice at geographical level. Several of its limitations drive researchers to use disease mapping methods -deemed as a valuable alternative. This work aims at exploring these techniques using - as a case of study- the gender differences in rates of hospitalization in elderly patients with chronic diseases.

**Methods:**

Design and study setting: An empirical study of 538,358 hospitalizations affecting individuals aged over 75, who were admitted due to a chronic condition in 2006, were used to compare Small Area Analysis (SAVA), the Besag-York-Mollie (BYM) modelling and the Shared Component Modelling (SCM). Main endpoint: Gender spatial variation was measured, as follows: SAVA estimated gender-specific utilization ratio; BYM estimated the fraction of variance attributable to spatial correlation in each gender; and, SCM estimated the fraction of variance shared by the two genders, and those specific for each one.

**Results:**

Hospitalization rates due to chronic diseases in the elderly were higher in men (median per area 21.4 per 100 inhabitants, interquartile range: 17.6 to 25.0) than in women (median per area 13.7 per 100, interquartile range: 10.8 to 16.6). Whereas Utilization Ratios showed a similar geographical pattern of variation in both genders, BYM found a high fraction of variation attributable to spatial correlation in both men (71%, CI95%: 50 to 94) and women (62%, CI95%: 45 to 77). In turn, SCM showed that the geographical admission pattern was mainly shared, with just 6% (CI95%: 4 to 8) of variation specific to the women component.

**Conclusions:**

Whereas SAVA and BYM focused on the magnitude of variation and on allocating where variability cannot be due to chance, SCM signalled discrepant areas where latent factors would differently affect men and women.

## Background

Geographical variability in healthcare utilization has become an important field within health services research in the last decades. Variation in medical practice studies aim to elicit systematic and unwarranted variability. As for the first goal, the efforts focus on ruling out randomness and on determining whether rates are consistent within a region and over time. In turn, drawing out unwarranted variability, differences in epidemiology (i.e., population's need) must be discarded.

With regard to the analytical approach, classically referred as Small Area Variation Analysis (SAVA) [1,2], it is based on the calculus of age and sex standardized utilization rates at population level derived from counts (procedures, hospital admissions), the estimation of several statistics of variation [3-7] and the representation of standardized utilization ratios on maps, describing patterns of "risk of utilization".

Studies based on SAVA have documented dramatic variations in the use of medical and surgical procedures across areas, but this analytical approach has some limitations in the estimation of systematic variation and, above all, the assessment of the underlying factors of such unwarranted variation. Among the most important ones we may highlight that age and gender are not always good surrogates of population's need [8], age groups or genders might have a differential behavior with regard to the endpoint of interest across regions [9], latent factors may not affect homogeneously to a given subgroup of population within and across regions [10], and finally, low rates or small populations might drive to imprecise results [6,11].

Some of these hindrances have been considered as a subject of study in the "disease mapping" framework, an epidemiological methodological approach used to describe and model geographical variation in disease risk and/or health outcomes, particularly the so called Shared Component Modelling (SCM), an extension of the most frequently used Besag, York and Mollié model (BYM) [12].

SCM is based on the idea that many diseases share common risk factors (i.e. latent factors); as a consequence, if similar patterns of geographical variation of related diseases can be identified, the evidence of real clustering could be more convincing. Later on, it was extended to more than two diseases [13], and showed to be more accurate than the use of independent disease-specific modelling. Subsequent works, that have compared the SCM with others, such as ecological regression or other multivariate conditional autoregressive models showed that its properties regarding precision estimates and goodness of fit, evidence it is a valuable extension of individual analysis [14-16]. Furthermore, it can be applied not only to related diseases [17], but also when analyzing deprivation domains [18], gender differences [16] or even comparing the evolution of the geographical gender differences over time [10]. The main idea of SCM is to borrow information from related diseases and health outcomes to strengthen inference, allowing to identify specific and shared (common to both) spatially-varying risk factors for each disease. In that way, it is possible to quantify the expected variability related to shared-risk factors and to tease out from the residual variations-specific patterns associated with each of the diseases under research.

The potential use of these shared component models in health services research is still unexplored. Our general aim is to take advantage of the methodological advances carried out in disease mapping, and transferring them into the health service research framework, to be able to derive findings that would have gone unnoticed otherwise. For this purpose, we applied shared component analysis to model hospital admission rates by chronic-disease in elderly male and female, comparing results with classical SAVA and BYM.

Our hypothesis postulates that, regardless of the actual differences in global rates between genders, a common pattern of variation is expected to explain most of the spatial variability; this hypothesis would entail that differences in the pattern of utilization by gender are constant across areas. Otherwise, discrepant patterns will allow us to identify those geographical areas in which latent factors like morbidity, socioeconomic status or health care policies have a differential behavior in men and women. These discrepant areas would deserve further analysis, under the assumption that those latent factors could explain part of the observed differences in hospitalization patterns.

## Methods

### Database, small geographic areas and procedures under study

We used data from the Atlas of Variations in Medical Practice in the Spanish National Health System (NHS) [19], a research project designed to inform Spanish decision-makers on differences in such parameters as hospital admissions or surgery for specific conditions across geographic areas (see: http://www.atlasvpm.org). The Spanish Atlas emulates the Dartmouth Atlas of Health Care Project [20]. Hospital Discharge Administrative Databases in 2006 (calendar year), with additional data from day-case surgery registries, were used to build the numerator of the rates. These administrative databases produced by every acute care hospital in the Spanish NHS, provide the following information from every single admission: age, gender, admission and discharge dates, postal codes identifying the patient's area of residence, and diagnosis and procedure codes [International Classification of Diseases 9^th ^revision Clinical Modification codes (ICD9CM)]. The postal code was used to assign every admission to the Healthcare Area where the patient lives.

Chronic disease was identified by means of the Chronic Condition Indicator (CCI) developed by the Healthcare Cost and Utilization Project (HCUP), a cooperative project sponsored by the United States Agency for Healthcare Research and Quality (AHRQ) [21]. A chronic condition is defined as a condition which lasts 12 months or longer and meets one or both of the following criteria: it places limitations on self-care, independent living, and social interactions, and/or it results in the need for ongoing intervention with medical products, services, and special equipment [22]. The identification of chronic conditions is based on all 5-digit ICD-9-CM codes, and assigns each case to one of the 18 categories that define the body system indicator (BSI). In this work, all 2006 hospital admissions corresponding to people aged 75 years and over with a main diagnosis of chronic disease, were considered.

Denominators to calculate population rates came from the 2006 Spanish National Institute of Statistics' Municipal Register of Inhabitants. The small geographic areas corresponded to the Healthcare Areas defined by the Health Departments of 16 out of the 17 Autonomous Regions participating in the Atlas Project -up to 180 geographical healthcare units. The expected number of cases per health unit, namely e_i _for the i-th area, was estimated separately by gender, using the rate for the whole region and the population at risk within the healthcare unit. It represents the number of admissions that would have been observed in the health unit under the hypothesis of constant rate across the whole region.

### Statistical Analysis

Statistics of variation (and their confidence intervals) such as the Extremal Quotient (EQ), the regular and weighted Coefficients of Variation (CV and CV_w_) [2], the Systematic Component of Variation (SCV) [3], and the Empirical Bayes statistic (EB)[7] were used to quantify variability. A previous work provided details on the properties of these statistics [7].

Three different approaches were used to model geographical variation of admissions for chronic diseases in men and women; the classical approach in SAVA studies, which estimates the utilization ratio for each gender; BYM which accounts for spatial autocorrelation and it was also applied separately to both genders; and, SCM which analyzes jointly both.

#### Classical small area analysis

This method compares the observed to the expected number of admissions per area assuming independence among areas, only using information referred to a particular area. The quotient of the observed (o_i_) to the expected (e_i_) number of cases, named Indirect Utilization Ratio (IUR_i _= o_i_/e_i _for the *i-th *Healthcare Area), is usually used to estimate and graph variation. This is equivalent assuming that the number of cases o_i _follows a Poisson distribution with mean e_i_ρ_i_, where ρ_i _denotes the underling risk parameter for the *i-th *area, and its estimate is derived using maximum likelihood for the saturated model. Significance for these estimates is derived using the exact method.

#### BYM modelling

The spatial model proposed by Besag, York, and Mollié [12] -which uses the so-called "local smoothing" due to "borrowing-strength" of neighboring areas - takes advantage of the knowledge of the spatial structure of the data, producing more stable estimates [23-25].

In the first level of the hierarchy, it assumes the same Poisson distribution as the SAVA model, o_i _~Poisson(e_i_ρ_i_), whereas in the second level of the hierarchy, instead of considering ρ_i _as a parameter to be estimated, it considers ρ_i _as a random variable whose logarithm is the sum of a constant termα α plus two random variables: the first one (u_i_) with a conditional autoregressive Gaussian structure (CAR normal distribution), and the second one (v_i_) with an exchangeable model.

That is, u_i _~CARNormal(W, $\tau_{\text{u}}=1/)\sigma_{\text{u}}^{2}$), with W the matrix representing the neighborhood structure (here two areas are assumed as neighbors if they share a common boundary) and τ_u _and $\sigma_{\text{u}}^{2}$ representing the precision and the conditional spatial variability respectively. Likewise, v_i _~N(0,$\tau_{\text{v}}=1/)\sigma_{\text{v}}^{2}$), with $\sigma_{\text{v}}^{2}$ representing the unstructured variability. From this model, the percentage of variability attributable to the spatial dependence can be derived from the quotient ${\text{s}}_{\text{um}}^{2}/)\left({\text{s}}_{\text{um}}^{2}+\sigma_{\text{v}}^{2}\right)$, where ${\text{s}}_{\text{um}}^{2}$ is the marginal spatial variance, ${\text{s}}_{\text{um}}^{2}=\sum_{\text{i}}{\left({\text{u}}_{\text{i}}-\bar{\text{u}}\right)}^{2}/)\left(\text{n}-1\right)$, being n the number of areas. To estimate the parameters of the model, two approaches can be conducted: the Empirical Bayes approach [26,27] via PQL methods[28] or the Full Bayes approach [24], which allows us to obtain the posterior distribution of the random variables, and the posterior probability maps - used as significance maps- representing Pr(ρ_i _> 1| data). Details on the model specification and its interpretation are provided in Additional file 1.

#### Shared component modelling (SCM)

In this work, SCM adopts some of the base specifications given in Knorr-Held and Best [29] and those implemented in Richardson [10]. It assumes that the area-specific hospital admission relative risks depend on a shared latent component common to men and women, plus additional latent components specific to each gender. These latent components act as surrogates for unmeasured hospital admissions risk factors that affect both or only one of the genders, respectively.

This model considers the same first level for each dataset as previous models

$$o_{1i}\sim Poisson\left(m_{1i}=e_{1i}\rho_{1i}\right);o_{2i}\sim Poisson\left(m_{2i}=e_{2i}\rho_{2i}\right)$$

$$\log\left(m_{1i}\right)=\log\left(e_{1i}\right)+\alpha_{1}+\mu_{1i};\;\log\left(m_{2i}\right)=\log\left(e_{2i}\right)+\alpha_{2}+\mu_{2i}$$

where *o*_1*i*_, *o*_2*i *_are the observed number of admissions by chronic diseases for men and women respectively, *e*_1*i*_, *e*_2*i *_*i *= 1,...,*n *the expected number of cases for both datasets and *α*, the intercept. In this model the spatial structure is introduced in a log scale by the joint structure of *μ*_1*i *_and *μ*_2*i*_

$$\mu_{1i}=\lambda_{i}\delta +\varphi_{1i};\;\;\;\mu_{2i}=\left(\lambda_{i}∕\delta \right)+\beta_{i}+\varphi_{2i}$$

where *λ_i _*represents the shared spatial pattern common for both datasets and *β_i _*represents the differential spatial pattern of women with respect to men. φ_1i _and φ_2i _are the residual terms to account for heterogeneity that may be left in the risk distribution after including the other terms in the model, and δ as the scaling parameter.

This Bayesian approach assumes that all parameters and random effects are unknown quantities that required the specification of the prior distribution. For this purpose we followed Wakefield, Best, and Waller recommendations [23], with only small variations to cope with this specific case. Regarding the random vectors **λ, β, φ_1 _**and **φ_2_**, the specifications are as follows. For the common spatial pattern given by **λ**, as well as for the discrepant component, a spatially structured distribution was adopted, **λ**~CARNormal(**W**, *τ_λ_*); **β**~CARNormal(**W**, *τ_β_*). For **φ_1_** and **φ_2 _**multivariate normal distributions N(0, τ_ϕ1_I) and N(0, τ_ϕ2_) where assumed with τ_ϕ1_and τ_ϕ2_as the precision parameters. Finally, the hyperprior specifications for the parameters were α's ~ dflats(), log(δ)~ N(0, 0.2), and τ's ~ Gamma(0.5, 0.0005). For this model, and equivalently to the BYM, the proportion of variability explained by each component for both datasets was derived from the empirical variances. Details on the model specifications are provided in Additional file 1.

Bayesian models' inference was made by using Markov Chain Monte Carlo (MCMC) simulations on the software R, version 2.9.2 via the library R2WinBUGS [30], which connects with the software WinBUGS [31]. To achieve convergence, 100,000 iterations keeping every 10^th ^were used after a burn-in period of 50,000. The classical diagnostic methods -Brooks and Gelman statistic [32], and sequential and autocorrelation graphs- were used to assess convergence. The Deviance Information Criterion (DIC) proposed by Spiegelhalter was used to compare models [33].

A Bayesian sensitivity analysis with various prior and hyperprior specifications, and the most frequently used distributions [34], was carried out. For prior distributions on **λ **and **β**, we compared exchangeable (normal independent) distributions with the assumed CARNormal. For hyperprior specification on the variances ($\sigma_{\text{1}}^{2}=1/)\tau_{\text{i}}$_i_, with τ_i _as the precision parameters above described), we compared the assumed inverse-gamma (0.5, 0.0005) with other three specifications, each one from a different family: a uniform on a wide range (U(0,100)) for σ_i_, an inverse-gamma (0.01, 0.01) for σ_i_^2^, and a half-normal prior density for σ_i _(Normal(0, τ = 0.01) I_(0, ∞)) _Finally, for the delta parameter, the assumed N(0, 5.5) on the log-scale was compared with the uniform assumption (U(0.5,2)) already used in other works [16]. Details of the sensitivity analysis and its results are given in Additional file 2.

## Results

The study setting, consisting of 180 healthcare units which account for 86% of the 2006 Spanish population, includes a total of 3,195,253 inhabitants aged 75 and over, among whom 62% are women. Table 1 shows a description of the population at risk, the admission rates by chronic diseases and the statistics of variation by gender. Men were more hospitalized than women, with a median rate of 21.4 per 100 inhabitants (interquartile range: 17.6 to 25.0) as compared to 13.7 per 100 (interquartile range: 10.8 to 16.6) in women. The relative variability among areas was very similar in both genders, with a ratio of 2.5 between the 95-th and the 5-th quantiles. Compared to the variability reported for hip fracture admission rates, frequently used as a standard of low variation, the CV, SCV and EB showed low to moderate geographical variability.

**Table 1:** Chronic disease admission rates and statistics of variation, by gender

	Men		Women	
	**Total**	**Median per area (IQ)**	**Total**	**Median per area (IQ)**
	
**Counts**	263,147	1125 (663 to 1976)	275,211	1108 (642 to 2021)
**Population**	1,227,278	5541 (3208 to 8940)	1,967,975	8733 (4995 to 14393)
**Rate**	21.44%	21.43 (17.56 to 25.01)	13.98%	13.70 (10.80 to 16.55)
**Variation Statistics**				
	EQ_5-95 _= 2.52 (2.32 to 2.92)		EQ_5-95 _= 2.55 (2.23 to 3.24)	
	CV = 0.27 (0.25 to 0.31)		CV = 0.30 (0.27 to 0.33)	
	CV_w _= 0.26 (0.24 to 0.31)		CV_w _= 0.29 (0.25 to 0.32)	
	SCV = 0.07 (0.06 to 0.10)		SCV = 0.09 (0.07 to 0.12)	
	EB = 0.07 (0.06 to 0.10)		EB = 0.10 (0.07 to 0.12)	

IQ: Interquartile Interval; EQ: Extremal Quotient; CV: Coefficient of Variation; CVw: Weighted Coefficient of Variation; SCV: Systematic Component of Variation; EB: Empirical Bayes statistic.

**Figure 1:**
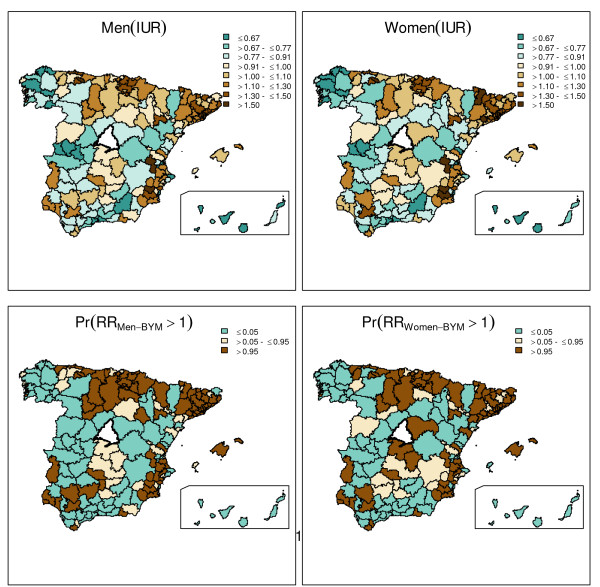
**Gender differences in the risk of admission**. Maps at the top show the utilization ratios estimated by using the classical model. Maps at the bottom show the estimation by using BYM posterior probability of a risk being above 1. Dark brown color in the posterior probability maps represents areas where the probability of having a relative risk of admission higher than 1 is above 0.95.

The geographical representation of the Indirect Utilization Ratio derived using the classical method (quotient of observed to the expected cases according to population at risk) is given at the top of Figure 1, which shows that there are regions at the north and the east part of the map which systematically have higher admission ratios, both in men and women, whereas the opposite occurs in some north and central east regions.

In turn, BYM provided practically the same point estimates for the risk of admission by area than the classical method, being the Pearson correlation between both methods of 0.99 for both genders, as expected given the large population size and frequency of the phenomenon studied. Model results are presented in Table 2, showing a fraction of variability attributable to spatial correlation of 71% for men and 62% for women, indicating the presence of a strong spatial pattern in both cases. From the variance component estimates it can be said that the global variability for men and women is quite similar, slightly higher in women though, in agreement with the statistics of variation. As for the whole density distribution provided by BYM, Figure 1 summarizes the probability of a risk of hospitalization being above 1. The figure shows a similar pattern for men and women, with higher risk of admission in the north and east and lower in the west.

**Table 2:** BYM modelling: results by gender

	Men Median (CI_95%_)	Women Median (CI_95%_)
**Parameter estimates**		
Unstructured variance($\sigma_{\text{h}}^{2}$)	0.022 (0.004,0.038)	0.037 (0.022,0.054)
Marginal Spatial variance($\sigma_{\text{u}}^{2}$)	0.054 (0.036,0.073)	0.060 (0.040,0.080)
**Fraction of variability explained**		
Spatial fraction	71.2% (50.1,94.5)	61.7% (45.2, 77.2)
**Model fit comparison criteria**		
DIC (Total DIC = 3888.07)	1942.81 (p_D _= 174.90)	1945.26 (p_D _= 175.721)

CI: Confidence Interval, type I error = 5%; DIC: Deviance Information Criterion

**Figure 2:**
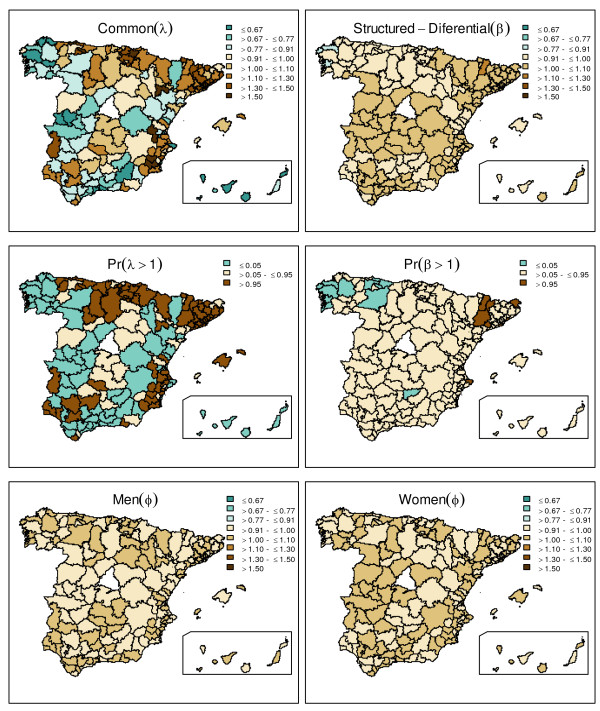
**Gender differences in the risk of admission: shared and differential components**. Map representing the posterior median of the shared spatial component is shown at the top left; whereas female-male differential spatial component is mapped at the top right. At the middle row, posterior probabilities for a risk being above 1 are shown for both, shared and differential components. Unstructured posterior median for the specific-gender components is shown at the bottom row. Dark brown color in the posterior probability maps represents areas where the probability for each component (common e^λ ^and structured discrepant e^β^, respectively), of having a relative risk of admission higher than 1 is above 0.95.

Finally, results derived from the SCM are shown in Table 3 and Figure 2. About 99.3% (CI95%: 97.4 to 99.8) of the spatial variation in men was captured by the shared term (λ), leaving only 0.7% (CI95%: 0.2 to 2.5) of the variability for the specific pattern of males. This shared term captured slightly less of the total spatial variation in women (94.2%; CI95%: 91.7 to 96.4), leaving a 5.8% (CI95%: 3.6 to 8.3) for the specific female component, which is mainly spatially correlated (a 4.2% out of the 5.8%). Hence, most of the risk was partitioned into the shared component, suggesting a weak residual signal.

**Table 3:** SCM modelling: results by gender

	Men	Women
**Fraction of total variations**		
% shared component (λ)	99.32% (97.45 to 99.82)	94.24%(91.68 to 96.37)
% specific component	0.68%(0.17 to 2.55)	5.76%(3.63 to 8.32)
Unstructured (*ϕ_1_, ϕ_2_*)	0.68%(0.17 to 2.55)	1.61%(0.23 to 4.38)
Spatially structured (β)		4.15%(1.73 to 6.70)
**Variance Components**		
Specific unstructured ($\sigma_{\phi }^{2}$)	0.0005 (0.0001 to 0.0019)	0.0015 (0.0002 to 0.0039)
Common spatial ($\sigma_{\chi }^{2}$)	0.0810 (0.0158 to 0.0865)	
Female specific spatial ($\sigma_{\beta }^{2}$)	0.0038 (0.0016 to 0.0062)	
Delta coefficient (δ)	0.967 (0.939 to 0.997)	
**Model fit comparison criteria**		
DIC (p_D_)	3845.7 (p_D _= 301.21)	

DIC: Deviance Information Criterion

A comparison between SCM and BYM in terms of goodness of fit, showed that SCM is superior (DIC_BYM_-DIC_SCM _= 43). A comparative analysis of the precision of the relative risk estimates, studied via the standard deviation of the log-relative risks, yielded a relative mean reduction of uncertainty about 22% in SCM compared to BYM.

SCM results indicate that the discrepancies between genders are small in the particular case of chronic diseases, but still it is of interest to allocate them. The first row in Figure 2 shows both the spatially structured common component (posterior median estimates of e^λi^) as well as the spatially structured discrepant one for females (e^βi^) using the posterior median estimates. Maps in the second row plot the probabilities for these risks of hospitalization being above 1. The shared component detects two noticeable clusters in the north and north-east showing higher common risks, apart from some high risk sparse regions in the south and south-east, all in agreement with previous models. The spatially structured discrepant pattern is much smoother, but still depicts slightly higher hospitalization risks for females in the centre part of the country, which are more marked in some north-east regions, in contrast to the lower risks in the north-west. Last row of the figure shows the unstructured specific pattern for males and females. It shows a very smooth pattern, given the low proportion of variability they explain.

Sensitivity analysis for SCM (Additional File 2) showed that: a) the choice of different spatial and non-spatial priors did not affect estimates; b) model comparison showed better DIC for models accounting for spatial correlation; and c) as for hyperprior distributions, uniform, half-normal, and inverse-gamma (0.5, 0.005) hyperpriors led to equal results, whereas inverse-gamma (0.01, 0.01) slightly biased some of the variance components, although not affecting final risk estimates.

Regarding the hospitalization relative risk estimates for men and women, the SCM model provides global results practically equivalent to both the BYM and classical methods, with a correlation between models above 0.99 in both cases.

## Discussion

In this study, both, classical SAVA and Bayesian techniques (BYM and SCM) have been used to elicit systematic and unwarranted gender differences in hospitalization for chronic conditions in elderly people. Actually, men with chronic conditions were more likely to be admitted than women (21.4 versus 13.9 admissions per 100 inhabitants).

The three methods provided different and, ultimately, complementary information upon this variation: SAVA showed that variation in men was slightly lower than that observed in women, but not negligible in either case, according to the EB statistic (table 1). In turn, BYM showed a reliable pattern of geographical clustering in the risk of hospitalization, taking into account uncertainty in each area (Figure 1). And eventually, SCM as our hypothesis postulated, drew up that the vast majority of the gender difference was constant across healthcare areas. And most relevant, only a 5.8% of the variation found a discrepant geographical pattern.

Up to now, most of the works based on aggregated data, and devoted to assess the geographical variation in utilization or health outcomes, carried out separate analysis for men and women. Using the classical methodology or standard Poisson regression assuming independence among areas, they estimated specific rates or ratios that allow comparison between genders [9,35-37]. The use of classical methods can be adequate in many contexts, but it is well known that when utilization rates are low, or when the geographical areas are low populated (where the risk of intra-area heterogeneity is larger), the instability of these indicators may produce misleading results [11]. Moreover, from an inferential standpoint, the strategy of a separate analysis does not help to understand underlying factors that might explain the difference between genders.

In our case-study, the use of this classical methodology allowed to observe that elderly men do have higher hospitalization rates than women in chronic conditions, that spatial variability was low to moderate in both cases, and that the geographical distribution of the utilization pattern was very similar.

Some of the deficiencies related to the instability of the estimates in the classical SAVA approach can be overcome by using the Bayesian hierarchical models proposed within the disease mapping framework. They take into account many nonstandard features in ecologic data such as strong patterns of dependence as well as a considerable level of noise [14]. Thus, the use of BYM to assess variation in medical practice would provide more reliable estimates of the hospital utilization patterns for infrequent events as wells as giving a quantification of the spatially correlated variability. It also yields probability maps that use not only point estimates for each region but also the whole posterior distribution (i.e. a representation of the statistical significance of the finding) leading to a more complete picture of the underlying utilization pattern [24].

In our case-study, BYM added, to the classical approach, information about the strong spatial pattern for both men and women, and provided the probability maps which allocated high-utilization areas in men and women suggesting similar patterns for both.

None of the aforementioned two approaches (SAVA and BYM) takes advantage of the fact that many risk factors, diseases, utilization patterns or health outcomes might share similar geographical patterns. If so, like it would be the case of gender disparity studies, joint modelling may lead to improved inference by reducing the number of alternative explanations for the observed variability [14]. Abundant disease mapping literature has been recently aimed to strengthen inference borrowing information from related factors. Developments pointed out that the SCM used in this work offers a significant improvement over individual BYM [17], and performs slightly better than other multivariate models [14].

In our case-study, we found that the model achieved considerable improvement both in terms of DIC (i.e. goodness of fit) and in getting more precise estimates of relative risks of hospitalization. Using the variance partitioning, the model also found high similarity in the pattern of hospitalization between men and women. And finally, it allowed to signal those regions in which disparities among genders were higher, such as those at the north-east with particularly lower rates in women.

The latter is precisely the most interesting property of SCM. The technique elicits discrepant areas, those where latent factors are affecting differently to men and women in the risk of hospitalization. Thus, this approach improves inference and may help in gaining further insight into the true underlying factors that are relevant to each specific gender. In this particular work, it could be hypothesized that the unmeasured factors expected to adopt a similar distribution between both genders are: time-distance to the referring hospital, socioeconomic gradient within the area or supply of primary care physicians. In turn, differences in morbidity or differences in the propensity to be referred to a hospital were able to be hypothesized as latent factors expected to affect differentially to men and women.

This SCM property would, eventually, have other potential applications in health services research, a field of knowledge challenged by the need of the adoption of new methodologies [38,39], and particularly, in the study of the geographical variations in medical practice. In addition to the study of gender inequalities in many domains (e.g., access to coronary revascularization, mental health unplanned admissions, knee replacement, avoidable hospitalizations, etc.), SCM could be used when analyzing healthcare adequacy to population's needs (e.g., Acute Myocardial Infarction rates vs coronary revascularization rates), technology substitution phenomenon (e.g., rates of conservative *versus *non-conservative mastectomy), alternative strategies of care at population level (e.g. defined-daily-doses of psychiatric drugs vs rates of mental health hospitalization in short-term units) or sub-optimal quality of care (e.g. knee replacement rates versus knee prosthesis revision rates).

Finally, to properly interpret and use SCM results, several caveats should be pointed out. SCM improves the ability of SAVA or traditional disease mapping Bayesian techniques in terms of inference; however, because of the nature of ecologic studies, caution is still needed when attributing variation to a specific cause. As an example, and out of the scope of this work, morbidity at population level should have been modelled, [40] to rule out this factor as an alternative explanation for the observed differences.

At a different point, it is worth noticing that although SCM improves the performance of classical techniques by smoothing the effect of small areas, extreme heterogeneity in population structure and size might still affect the estimates; ultimately, misleading towards attributing variation to a specific cause -differential gender access in our example-, when the underlying reason is on the differences in the population structure [41].

Finally, variability studies showed us that variation is expected to be a local phenomenon. SCM, like the other Bayesian techniques, models the "vicinity effect" borrowing information from the counts in the neighbored areas, smoothing the estimated variance. So, factors like different practice style or different strategies of admission between genders, which are expected to explain variation across areas, have been also smoothed. If these factors were highly predictive in producing unwarranted variability, the obtained results would have under-estimated the actual variation.

## Conclusion

As the conclusion of this empirical study, it could be stated that, whereas SAVA and BYM focus on the magnitude of the variability and on allocating where this variation cannot be due to chance (being the latter more accurate in the estimates because it accounts for spatial autocorrelation), SCM signals those discrepant areas where latent factors are affecting differently to men and women in the risk of hospitalization, improving the inferential capacity of the other techniques.

## Competing interests

The authors declare that they have no competing interests.

## Authors' contributions

All the authors are guarantors of the study, had full access to all the data, and take responsibility for the integrity and the accuracy of the analysis and results. BI, JL specifically contributed to the study analysis. SP and EBD interpreted the results. BI, JL and EBD drafted the article. All the authors read and approved the final manuscript.

## Pre-publication history

The pre-publication history for this paper can be accessed here:

http://www.biomedcentral.com/1471-2288/11/172/prepub

## Supplementary Material

Additional file 1 **Models description**. Detailed description on the assumptions for each model, the estimation procedures and the outcomes that can be derived. Click here for file

Additional file 2 **Shared component modelling sensitivity analysis**. Description on the sensitivity analyses conducted to check the estimations robustness. Click here for file
