# Supplementary material for: Shared component modelling as an alternative to assess geographical variations in medical practice: gender inequalities in hospital admissions for chronic diseases
Source: BMC Med Res Methodol. 2011 Dec 21;11:172. doi: 10.1186/1471-2288-11-172 (PMC3273448; doi:10.1186/1471-2288-11-172)
Supplement: Additional file 1 — Models description. Detailed description on the assumptions for each model, the estimation procedures and the outcomes that can be derived. [file 1471-2288-11-172-S1.DOC]

**Additional file 1**

Characteristics of each model: assumptions, estimation procedures and the outcomes that can be derived from each of them

|  |  | **MALES** | **FEMALES** |
| --- | --- | --- | --- |
| **CLASSICAL MODEL** | |  |  |
| **1^st^: Assumptions for the first level of the hierarchy** | | | |
|  |  | $o_{1i}$~$Poisson(m_{1i}=e_{1i}\rho_{1i})$ | $o_{2i}$~$Poisson(m_{2i}=e_{2i}\rho_{2i})$ |
| **2^nd^: Assumptions for the second level of the hierarchy** | | | |
|  |  | Independence between areas | |
|  |  | Independence between genders | |
| **3^rd^:Estimation** | | Maximum Likelihood: $\rho_{1i}$: IUR_1i_=o_1i_/e_1i_ | $\rho_{2i}$:IUR_2i_=o_2i_/e_2i_ |
| **4^th^:Outcomes** | |  | |
|  |  | **Estimates:** For $\rho_{1i}$, for$\rho_{2i}$, for rates, all with CI_95%_, | |
|  |  | **Maps:** Maps with the estimates | |
|  |  | **Variation:** Statistics of variation: EQ, CV, CV_w_, SCV, EB | |
| **BYM MODEL** | |  |  |
| **1^st^: Assumptions for the first level of the hierarchy** | | | |
|  |  | $o_{1i}$~$Poisson(m_{1i}=e_{1i}\rho_{1i})$ | $o_{2i}$~$Poisson(m_{2i}=e_{2i}\rho_{2i})$ |
| **2^nd^: Assumptions for the second level of the hierarchy** | | | |
|  |  | Dependence between areas | |
|  |  | Independence between genders | |
|  |  | log(ρ_1i_)= α + u_1i_+v_1i_ | log(ρ_2i_)= α + u_2i_+v_2i_ |
|  |  | u_1i_~CARNormal(W,τ_1u_=1/σ_1u_^2^) | u_2i_~CARNormal(W,1/σ_2u_^2^) |
|  |  | v_1i_ ~N(0, τ_1v_=1/σ_1v_^2^) | v_2i_ ~N(0, τ_v_=1/σ_2v_^2^) |
| **3^rd^:Estimation** | | MCMC procederes | |
| **4^th^:Outcomes** | |  | |
|  |  | **Estimates:** Smoothed $\rho_{1i}$ $\rho_{2i}$P($\rho_{1i}$>1| data) P($\rho_{2i}$>1|data) | |
|  |  | **Maps:**$\rho_{1i}$,$\rho_{2i}$pattern & significance maps | |
|  |  | **Variation:** % variability attributable to the spatial dependence | |
|  |  | **Evaluation:**Convergence and DIC | |
| **SCM MODEL** | |  |  |
| **1^st^: Assumptions for the first level of the hierarchy** | | | |
|  |  | $o_{1i}$~$Poisson(m_{1i}=e_{1i}\rho_{1i})$ | $o_{2i}$~$Poisson(m_{2i}=e_{2i}\rho_{2i})$ |
| **2^nd^: Assumptions for the second level of the hierarchy** | | | |
|  |  | Dependence between areas | |
|  |  | Dependence between genders | |
|  |  | $\log\left(m_{1i}\right)=\log\left(e_{1i}\right)+\alpha_{1}+\mu_{1i};$ | $\log\left(m_{2i}\right)=\log\left(e_{2i}\right)+\alpha_{2}+\mu_{2i}$ |
|  |  | $\mu_{1i}=\lambda_{i}\delta+\varphi_{1i}$ | $\mu_{2i}=(\lambda_{i}/\delta)+\beta_{i}+\varphi_{2i}$ |
|  |  | $\mathbf{\lambda}$~CARNormal$\left(\mathbf{W},\tau_{\lambda}\right)$ | $\mathbf{\beta}$~CARNormal$\left(\mathbf{W},\tau_{\beta}\right)$ |
|  |  | $\mathbf{\varphi}_{1}$~** **N(0, τ_φ1_I) | $\mathbf{\varphi}_{2}$~** **N(0, τ_φ2_I) |
| **3^rd^:Estimation** | | MCMC procederes | |
| **4^th^:Outcomes** | |  | |
|  |  | **Estimates:** Smoothed $\rho_{1i}$,$\rho_{2i}$,P($\rho_{1i}$>1), P($\rho_{2i}$>1) | |
|  |  | **Maps:**$\rho_{1i}$,$\rho_{2i}$pattern & significance maps, common & discrepant maps | |
|  |  | **Variation:** % variability attributable to each component: | |
|  |  | **Evaluation:** Convergence and DIC | |

* Notation used in previous table

|  | **Specification** | **Explanation** |
| --- | --- | --- |
| **Common notation** | | |
|  | $o_{1i}$,$o_{2i}$ | Number of admissions in area i in males and in females |
|  | $e_{1i}$,$e_{2i}$ | Number of expected admissions in area i in males and in females, assuming common rates along the whole region |
|  | $\rho_{1i}$,$\rho_{2i}$ | Risk of admissions in males and females in area i, unknown, needs to be estimated |
| **Classic model** | |  |
|  | IUR_1i_, IUR_2i_ | Indirect Utilization ratios, which are the estimates for$\rho_{1i}$,$\rho_{2i}$ via Maximum-Likelihood IUR_1i_=o_1i_/e_1i_ |
|  | EQ, CV, CV_w_, SCV, EB | Statistics of Variation EQ: Extremal Quotient; CV: Coefficient of Variation; CVw: Weighted Coefficient of Variation; SCV: Systematic Component of Variation; EB: Empirical Bayes statistic. Higher values indicate higher variability. |
| **BYM model** | |  |
|  | u_1i_, u_2i_ | Random effects which model the spatial correlation in the risk of admission for males (u_1i_) and females (u_2i_). To do so, a conditional autorregresive distribution is assigned for each one, CARNormal(W,τ_1u_=1/σ_1u_^2^). If data are not correlated, the variance component estimate, σ_1u_, will be low |
|  | v_1i_,, v_2i_ | Random effects which have independent structure, N(0, τ_1v_=1/σ_1v_^2^). They need to be included together with the u_i_ random vector, otherwise we would be forcing spatial structure where it may not be present. |
|  | % variance spatially structured | =s_um_^2^/ (s_um_^2^ + σ_v_^2^), where s_um_^2^ is the marginal spatial variance, s_um_^2^=∑_i_(u_i_ -ū)2/(n-1), |
|  | P($\rho_{1i}$>1| data)  P($\rho_{2i}$>1| data) | The posterior probability for area i to have an admission risk higher than that for the whole region, given the data. These values are often used to derive probability maps, for which cut-off points 0.2 and 0.8 are used. |
|  | DIC | Deviance Information Criteria: it is used in the Bayesian framework to compare models. DIC=D+ p_D_, where D is the Deviance average and p_D_ the number of effective parameters. Models with smaller DIC are preferred. |
| **SCM model** | |  |
|  | $\mu_{1i},\mu_{2i}$ | In the log-scale, the relative risk of admission in area i for males and females compared to that of the whole region |
|  | $\lambda_{i}$ | The random effect assigned to area i, which represents the shared risk in males and females. We assumed it as spatially correlated. |
|  | δ | A parameter that allows for a different gradient on the shared component for males and females. |
|  | $\beta_{i},\varphi_{1i},\varphi_{2i}$ | The random effects assigned to area i, which depict differences between males and females with respect to the common pattern.$\varphi_{1i}$is referred to males and $\beta_{i}$and $\varphi_{2i}$are referred to females, $\beta_{i}$with spatial structure and $\varphi_{1i}$and $\varphi_{2i}$with an independent structure. |
|  | Partition of variance | The whole variability observed in males can be decomposed by that attributed to the common pattern and that specific to males. The same applies to females. |
|  | P($\rho_{1i}$>1| data), DIC | Idem explanation as for BYM model |
