# Supplementary material for: Shared component modelling as an alternative to assess geographical variations in medical practice: gender inequalities in hospital admissions for chronic diseases
Source: BMC Med Res Methodol. 2011 Dec 21;11:172. doi: 10.1186/1471-2288-11-172 (PMC3273448; doi:10.1186/1471-2288-11-172)

**Additional file 2**

**Sensitivity analysis for the Share Component Model**

**Specifications for priors and hyperpriors**

Table 1 shows the specifications that have been used in the sensitivity analysis. Model 1 has the specifications used in the model proposed and described in the main text. Models 2 and 3 consider different prior structures, but assume the same hyperpriors as Model 1. Models 3 to 7 assume the same prior structure, but change the hyperprior specifications.

With regard to changes in the prior specifications, Model 2 considers the common and specific patterns not spatially structured, including less parameters than in Model 1 (i.e., the random effect β is not present). Model 3 assumes a common pattern spatially structured, as Model 1, but assumes a discrepant pattern for both males and females, not spatially structured (as Model 2).

Concerning changes in hyperprior specifications, the precision terms in Model 1 were assigned a conjugate hyper-prior Gamma (0.5, 0.005), which is weakly informative, and has been widely used in the shared component modelling [10,17,18]. Specifications for Model 4 are based on the same gamma family for the precision parameters, but with different shape and scale (Gamma (0.01, 0.01)). Model 5 assumes a uniform distribution for σ_i_ on a wide range (U(0,100)). The uniform distribution U(0, A) for a large A was suggested by Gelman [34], who shows that for a finite but sufficiently large A, inferences are not sensitive to the choice of A, recommending the use of the uniform distribution on σ_i_, but not on σ_i_^2^. Model 6 uses a weakly informative half-normal prior density for σ_i_, that is, σ_i_ ~ Normal(0, τ= 0.01)I_(0, ∞)_). This assumption, still not widely used, has been proved to perform well in different contexts [34].

Finally, with regard to the specifications for other parameters, Model 7 assumes an informative prior for the delta parameter, specifically U(0.5, 2), instead of the assumed N(0, 5.5) on the log-scale for Model 1, following the specifications given elsewhere [16].

**Table 1 Specifications used for the sensitivity analyses**

|  |  |  | **Priors** | | | |  | **Hyperpriors** | | |
| --- | --- | --- | --- | --- | --- | --- | --- | --- | --- | --- |
|  |  |  | $\mathbf{\lambda}$ | $\mathbf{\beta}$ | $\mathbf{\varphi}_{1}$ | $\mathbf{\varphi}_{2}$ |  | **α´s** | **δ** | ** τ´s** |
|  | **Model 1** |  | CAR | CAR | N(0,τ_φ1_I) | N(0,τ_φ2_I) |  | dflats() | log**δ**~N(0,0.2) | Γ(0.5, 0.0005) |
|  | **Model 2** |  | N(0,τ_λ_I) | No$\mathbf{\beta}$ | = | = |  | = | = | = |
|  | **Model 3** |  | = | No$\mathbf{\beta}$ | = | = |  | = | = | = |
|  | **Model 4** |  | = | = | = | = |  | = | = | Γ(0.01,0.01) |
|  | **Model 5** |  | = | = | = | = |  | = | = | σ _i_~N(0,0.01) I_(0,∞)_ |
|  | **Model 6** |  | = | = | = | = |  | = | = | σ _i_~U(0,100) |
|  | **Model 7** |  | = | = | = | = |  | = | δ ~U(0.5, 2) | = |

Note: The symbol “=” represents those specifications that are equal to Model 1

**Results**

Table 2 shows the results of the models with respect to the fraction of the total variation obtained (in percentage). It suggests that the results do not vary at all when different specifications are used. The model that seems to change more is Model 2, which assumes independence in common and discrepant patterns, but differences are minimal and do not affect the interpretation of the results. A comparison of the DIC statistics between models with different prior specifications suggests that models accounting for the spatial correlation are better than those which do not (i.e., Model 2 and Model 3 have 7 to 9 points more than Model 1).

**Table 2**

**Fraction of total variation attributable to each component for Models 1 to 7**

|  | **DIC** |  | **Males** | |  | **Females** | | | |
| --- | --- | --- | --- | --- | --- | --- | --- | --- | --- |
|  |  |  | **Common** | ***Specific*** |  | **Common** |  | ***Specific*** | |
|  |  |  | $\mathbf{\lambda}$**(CI_95%_)** | ***φ_1_* (CI_95%_)** |  | $\mathbf{\lambda}$**(CI_95%_)** |  | **Structured**$\mathbf{\beta}$ | ***φ_2_* (CI_95%_)** |
| **Model 1** | 3846 |  | 99.3(97.4-99.8) | 0.7(0.2-2.5) |  | 94.2(91.7-96.4) |  | 4.2(1.7-6.7) | 1.6(0.2-4.4) |
| **Model 2** | 3853 |  | 96.9(93.5-99.7) | 3.1(0.3-6.5) |  | 97.0(93.5-99.8) |  | - | 3.0(0.2-6.5) |
| **Model 3** | 3855 |  | 97.9(94.0-99.7) | 2.1(0.2-6.5) |  | 96.0(93.4-99.5) |  | - | 4.0(0.5-6.6) |
| **Model 4** | 3847 |  | 97.8(96.3-98.7) | 2.3(1.3-3.7) |  | 92.5(89.3-95.5) |  | 3.2(1.8-5.2) | 4.3(1.6-6.4) |
| **Model 5** | 3850 |  | 99.7(97.9-100) | 0.3(0.0-2.1) |  | 92.1(89.0-95.1) |  | 4.5(2.3-7.0) | 3.6(0.1-5.6) |
| **Model 6** | 3842 |  | 99.7(97.8-100) | 0.3(0.0-2.2) |  | 92.1(89.2-95.0) |  | 4.4(2.1-6.8) | 3.6(0.4-5.6) |
| **Model 7** | 3848 |  | 99.3(97.6-99.8) | 0.7(0.2-2.4) |  | 92.5(89.5-95.5) |  | 4.0(1.8-6.5) | 3.5(0.5-5.4) |

Figure 1 shows the results of the risk estimates using Models 2 to 7 compared to Model 1. Each row gathers the comparison between the estimates obtained for the respective model (y-axis) and the estimates for Model 1 (x-axis). The first column shows the smoothed relative risks for males, the second one for females, the third one the common lambda component (in an exponential scale), and the last one the spatially structured discrepant component beta (also in an exponential scale). The results regarding the relative risks show that the points lie perfectly on the line of unity, showing perfect agreement between all models. For the case of the common pattern, it seems that models 2 and 3, which do not consider spatial correlation, differ slightly from the rest. This pattern is in line with the results obtained from the comparison of percentage of variation given in table 2, and with previous works [16], where differences were found in the results when spatial and non-spatial priors were compared. Finally, the structured differential component estimates, also lie on the diagonal regardless the hyper-prior specifications; nevertheless, a slight bias seems to appear in Model 4, which assumed a Gamma (0.01, 0.01) for the precision parameters. This result may be related to the recommendations given by Gelman [34], who pointed out that when a prior density Gamma(ε,ε) with ε tending to 0 is assumed, posterior inferences are sensitive to ε, and therefore appropriate values for ε need to be considered.

In summary, results of the sensitivity analysis suggest that the choice of hyper-prior has no effect on our main results.

**Figure 1**

**Relative Risks and common and differential parameters for the different prior and hyper-prior specifications**


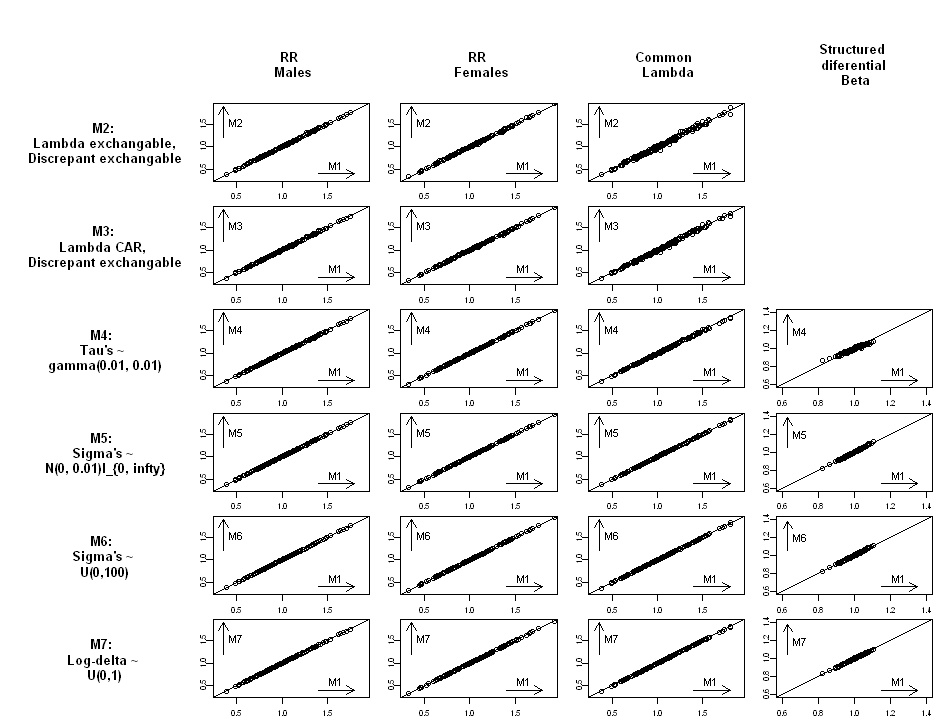

Supplement: Additional file 2 — Shared component modelling sensitivity analysis. Description on the sensitivity analyses conducted to check the estimations robustness. [file 1471-2288-11-172-S2.DOC]
